# Indoleamine-pyrrole 2,3-dioxygenase-1 (IDO-1) mRNA is over-expressed in the duodenal mucosa and is negatively correlated with serum tryptophan concentrations in dogs with protein-losing enteropathy

**DOI:** 10.1371/journal.pone.0218218

**Published:** 2019-06-10

**Authors:** Aarti Kathrani, Victor Lezcano, Edward J. Hall, Albert E. Jergens, Yeon-Jung Seo, Jonathan P. Mochel, Todd Atherly, Karin Allenspach

**Affiliations:** 1 Royal Veterinary College, Hatfield, Hertfordshire, United Kingdom; 2 College of Veterinary Medicine, Tuskegee University, Tuskegee, Alabama, United States of America; 3 Bristol Veterinary School, University of Bristol, Langford, Bristol, United Kingdom; 4 College of Veterinary Medicine, Iowa State University, Ames, IA, United States of America; University of Lincoln, UNITED KINGDOM

## Abstract

**Introduction:**

Dogs with protein-losing enteropathy (PLE) have decreased serum tryptophan concentrations, which may contribute to disease pathogenesis. Indoleamine-pyrrole 2,3-dioxygenase-1 (IDO-1) expression is associated with low serum tryptophan concentrations and is increased in the gastrointestinal tract of humans with inflammatory bowel disease (IBD). Therefore, the objective of our study was to determine if the mRNA expression of IDO-1 is increased in the duodenal mucosa of dogs with PLE as compared to dogs with chronic enteropathy (CE) and healthy dogs, and whether this expression is correlated with changes in serum tryptophan concentration.

**Methods:**

Our study was a retrospective study using archived paraffin-embedded duodenal biopsy specimens from 8 healthy Beagle dogs from the Iowa State University Canine Service Colony and 18 and 6 client-owned dogs diagnosed with CE and PLE, respectively at the Bristol Veterinary School. A novel RNA *in situ* hybridization (ISH) technology, RNAscope, was used to identify IDO-1 mRNA mucosal expression in duodenal tissues. An IDO-1 specific probe was hybridized onto 10 duodenal biopsy sections from each dog whereby RNAscope signal (mRNA expression) was quantified by a single operator using light microscopy.

**Results:**

Dogs with PLE had significantly higher mRNA expression of IDO-1 in the duodenal mucosa compared to healthy dogs (mucosal percentage IDO-1 positive: *P* = 0.0093, (mean ± S.D) control: 19.36 ± 7.08, PLE: 34.12 ± 5.98, average fold difference: 1.76 and mucosal IDO-1 H-score: *P* = 0.0356, (mean ± S.D) control: 45.26 ± 19.33, PLE: 84.37 ± 19.86, average fold difference: 1.86). The duodenal mucosal mRNA expression of IDO-1 was negatively correlated with serum tryptophan concentrations in dogs with PLE (mucosal IDO-1 H-score: Spearman’s rank correlation coefficient = -0.94, *P* = 0.0048).

**Conclusions:**

In conclusion, our study suggests that decreased serum tryptophan concentrations in dogs with PLE is associated with increased intestinal IDO-1 expression. Further studies are needed to determine potential inflammatory pathways responsible for increased expression of IDO-1 in the intestinal tract of dogs with PLE.

## Introduction

Indoleamine-pyrrole 2,3-dioxygenase-1 (IDO-1) is an enzyme expressed by cells of the innate immune system and acts as the interface with the adaptive immune system [1]. IDO-1 promotes immune tolerance by influencing T-cell proliferation and clonal expansion and has also been shown to have antimicrobial properties [1, 2]. Toll-like receptor activation as well as pro-inflammatory cytokines, such as interferon-gamma and tumor necrosis factor- alpha, may induce IDO-1 expression [1, 3]. Therefore, IDO-1 expression is increased in the gastrointestinal (GI) tract of both humans with inflammatory bowel disease (IBD) and animal models of colitis, due to overexpression of pro-inflammatory cytokines and over-activation of toll-like receptors [4–6]. IDO-1 is also the initial rate-limiting step in the pathway for the oxidation of tryptophan to kynurenine [7]. Therefore, increased IDO-1 expression is associated with lower serum tryptophan concentrations in humans with IBD due to increased tryptophan catabolism [8].

Recently, dogs with protein-losing enteropathy (PLE) have been documented to have significantly lower serum tryptophan concentrations compared to healthy dogs [9]. Tryptophan is a dietary essential amino acid in dogs and is important for protein synthesis as well as serving as a precursor for bioactive compounds, such as kynurenine, serotonin, melatonin and picolinic acid [7]. Endogenous tryptophan metabolites have been shown to protect from mucosal inflammation by maintaining gut immune homeostasis and microbial diversity in animal models of colitis [10, 11]. Furthermore, tryptophan-deficient mice showed more severe colitis when administered oral dextran sodium sulfate [12]. Hence, tryptophan is important in helping regulate beneficial physiological functions and reduce mucosal injury in the GI tract [13, 14]. Consequently, decreased serum tryptophan concentrations in dogs with PLE might contribute to or aggravate intestinal inflammation. Further studies are needed to determine the pathomechanism of decreased serum tryptophan concentrations and to provide insight into those treatment modalities likely to restore tryptophan concentrations in dogs with PLE.

Collectively, studies in humans with IBD and experimental models of colitis suggest that decreased serum tryptophan concentration in dogs with PLE might be due to increased intestinal IDO-1 expression causing increased catabolism of tryptophan. However, IDO-1 expression has not been previously assessed in the intestinal mucosa of dogs with PLE. Therefore, our study aimed to determine whether mRNA expression of IDO-1 is altered in the duodenal mucosa of dogs with PLE compared to dogs with chronic enteropathy (CE) and healthy dogs, and whether this expression correlated with serum tryptophan concentrations in dogs with PLE.

## Materials and methods

### Study design

We performed a retrospective study using archived paraffin-embedded duodenal biopsy specimens obtained from dogs with PLE or CE referred to the University of Bristol. Tissue specimens from three groups of dogs were studied: PLE (n = 6), CE (n = 18) and healthy controls (n = 8) comprising a population of young adult Beagle dogs at the Iowa State University.

### Retrospective study criteria for case selection

Six dogs diagnosed with PLE at the Bristol Veterinary School that previously had serum tryptophan concentrations measured as part of a separate study were specifically chosen [9]. Diagnostic evaluations performed on all 6 dogs included a complete blood count, serum biochemistry, serum cobalamin and folate concentrations, pre- or pre- and post- prandial bile acid concentrations, urine protein creatinine ratio, empirical deworming, trans-abdominal ultrasound examination and gastrointestinal endoscopy and histopathology of intestinal biopsies. Of the 6 dogs included in the PLE group, the following number had additional diagnostic procedures performed: pancreatic testing (canine pancreatic lipase immunoreactivity in 3 dogs and trypsin-like immunoreactivity in 3, basal cortisol concentration or ACTH stimulation test in 4 dogs, fecal parasitology using zinc sulfate flotation with centrifugation in 5 dogs, and fecal culture (for *Salmonella*, *Campylobacter* and *Clostridium difficile*) in 5 dogs. The albumin concentrations of these 6 dogs ranged from 13.7 to 19.8 g/L (laboratory reference range (32–38) with a median of 15.2 g/L.

The medical records at the Bristol Veterinary School were searched for dogs that had been diagnosed with CE but without biochemical abnormalities consistent with PLE (panhypoproteinemia with an albumin concentration of less than 32 g/L or solely hypoalbuminemia with a concentration of less than 25 g/L were used as exclusion criteria). All medical records then were reviewed by 1 of the authors (AK). Only dogs with a histologic diagnosis of chronic inflammatory enteropathy that had adequate and appropriate investigations to exclude other causes of chronic GI signs before histologic diagnosis were included. Diagnostic evaluations performed on all 16 dogs included a complete blood count, serum biochemistry and gastrointestinal endoscopy and histopathology of intestinal biopsies. Of the 16 dogs included in the CE group, the following number had the following diagnostic procedures performed: abdominal imaging with either trans-abdominal ultrasound examination in 14 dogs or computed tomography in 1 dog, pancreatic testing (canine pancreatic lipase immunoreactivity in 7 dogs and trypsin-like immunoreactivity in 8, basal cortisol concentration or ACTH stimulation test in 10 dogs, pre- or pre- and post- prandial bile acid concentrations in 7 dogs, fecal parasitology using zinc sulfate flotation with centrifugation in 5 dogs and fecal culture (for *Salmonella*, *Campylobacter* and *Clostridium difficile*) in 4 dogs.

The canine chronic enteropathy activity index (CCEAI) [15], which is based on the presence and severity of nine factors including attitude/activity, appetite, vomiting, stool consistency, stool frequency, weight loss, serum albumin concentrations, ascites and peripheral edema and pruritus was retrospectively calculated for each dog based on the history collected at admission and the referring veterinarian’s medical records.

Archived paraffin embedded duodenal biopsies were retrieved from the archive at Bristol Veterinary School from the selected cases and at least 10 serial sections, mounted onto glass slides from each dog were sent to Iowa State University for IDO-1 RNA *in situ* hybridization.

### Selection of control dogs

The research colony of the Iowa State University Center of Veterinary Medicine (8 female spayed Beagle dogs at 2 years of age) was used to obtain endoscopic intestinal biopsies from healthy control dogs. The dogs were housed in groups of 4 per kennel with access to outside and behavioral enrichment with training provided by veterinary students three times per week. They were fed a commercial dog food twice per day with ad libitum access to water and each dog was assessed daily for health by a veterinarian. The dogs were assessed as clinically healthy and had a complete blood count, serum biochemistry, urinalysis, and fecal parasitology performed, which were all normal. Gastrointestinal endoscopy was performed under general anaesthesia and buprenorphine was administered as an analgesic; all procedures were performed at the veterinary hospital at the Iowa State University. All dogs were adopted after the study.

### RNA *in situ* hybridization

RNA *in situ* hybridization (ISH) allows the detection of specific RNA sequences with cellular resolution within tissue architecture on routine formalin-fixed, paraffin-embedded tissue specimens. Specifically, a novel RNA ISH technology, RNAscope (ACD Biotech, Newark, CA), which provides single-molecule visualization while preserving tissue morphology was used for diagnostic purposes. Two slides with 5 or more duodenal biopsies per dog each were hybridized with the IDO-1 mRNA specific probes and examined, including use of positive controls for each hybridization using Ubiquitin C. Preparation and hybridization of the slides was done in accordance to the user manual protocol provided by Advanced Cell Diagnostics (ACD) Biotech.

### Visualization and image capture

RNAscope signals were first visualized by using a 20x Plan N lens on an Olympus BX40 microscope (Olympus Optical Co., LTD, Japan) and photographed with an Olympus DP27 camera (Olympus Optical Co., LTD, Japan). Ten images were obtained from different representative fields per glass slide. A representative mucosal field was defined as containing 3 or more duodenal biopsy specimens containing at least 3 contiguous villi for diagnostic evaluation [16].

### Quantitative analysis of IDO-1 expression

Quantitative assessment of IDO-1 mRNA expression was performed utilizing the images captured and was performed by a single operator (VL). The quantitative assessment consisted of average mucosal IDO-1 copies per cell, average mucosal IDO-1 area per cell, mucosal percentage IDO-1 positive and mucosal IDO-1 H-score for all dogs. H-scores were calculated using RNAscope technology and digital quantitation by HALO using the data analysis guide provided by ACD Biotech.

### Serum tryptophan concentrations in dogs with PLE

Serum collected at the time of diagnosis from the 6 dogs with PLE had previously been analyzed for tryptophan using an automated high-performance liquid chromatography amino acid analyzer as part of a previous study [9]. None of the dogs with CE or healthy controls had serum tryptophan measurements performed.

### Ethical considerations

Archived paraffin-embedded duodenal biopsies were used in this study and the University of Bristol granted ethical approval for the study (VIN/18/014). Iowa State University granted ethical approval for the use of paraffin-embedded duodenal biopsies from healthy colony Beagle dogs (9-17-8605-K).

### Data analysis and statistics

One-way analysis of variance (ANOVA) was used to assess differences in mean values for the mRNA expression of IDO-1 in the duodenal mucosa (average mucosal IDO-1 copies per cell, average mucosal IDO-1 area per cell, percentage mucosal IDO-1 positive and mucosal IDO-1 H-score) between the three groups of dogs (PLE, CE and Control). If significant difference was identified among the groups, post-hoc pairwise comparisons test was performed using Tukey's HSD (Honestly Significant Difference). The Spearman’s Rank correlation test was used to determine if there was a significant correlation between mRNA expression of IDO-1 in the duodenal mucosa and serum tryptophan concentrations in dogs with PLE. Analyses were performed using R software version 3.5.2 (R Foundation for Statistical Computing, Vienna, Austria) and IBM SPSS Statistics Version 24. Statistical significance was defined as *P* < 0.05.

## Results

### Dogs

Six dogs with PLE were included; 4 female neutered and 2 male neutered dogs, with a median age of 8.0 years and a range of 3.5 to 12. There were 2 Labrador retrievers and one each of the following breeds: Japanese Akita, Staffordshire bull terrier, border terrier and Jack Russell terrier. The median CCEAI was 9 (severe disease activity) with a range of 4 to 15. Two dogs were diagnosed with lymphoplasmacytic and eosinophilic enteritis, three with lymphoplasmacytic enteritis and one with lymphoplasmacytic, neutrophilic and eosinophilic enteritis.

Eighteen dogs with CE were included; 10 female neutered, 7 male neutered and one male intact dog, with a median age of 6.7 years and a range of 0.8 to 11. There were 8 German shepherd dogs, 2 Border collies, 2 Boxers and one each of the following breeds: West Highland white terrier, Beagle, Siberian husky, Chihuahua, Border terrier and Cavalier King Charles spaniel. The median CCEAI was 8.5 (moderate disease activity) with a range of 3 to 13. Seven dogs were diagnosed with eosinophilic enteritis, 4 dogs with lymphoplasmacytic, eosinophilic and neutrophilic enteritis, 3 dogs with lymphoplasmacytic and eosinophilic enteritis, 2 dogs with lymphoplasmacytic enteritis, one with lymphocytic enteritis and one with plasmacytic enteritis.

There were no significant differences in age or CCEAI between the PLE and CE groups (*P* >0.204).

Eight healthy Beagle control dogs were included; all were female and 2 years of age.

### IDO-1 mRNA

One-way ANOVA comparing the mRNA expression of IDO-1 in the duodenal mucosa (average mucosal IDO-1 copies per cell, average mucosal IDO-1 area per cell, percentage mucosal IDO-1 positive and mucosal IDO-1 H-score) between the three groups of dogs (PLE, CE and Control) showed a significant difference in percentage mucosal IDO-1 positive mRNA and mucosal IDO-1 H-score between the three groups (*P* = 0.0123 and 0.0435, respectively). However, average mucosal IDO-1 copies per cell and average mucosal IDO-1 area per cell were not significantly different between the three groups.

**Fig 1:**
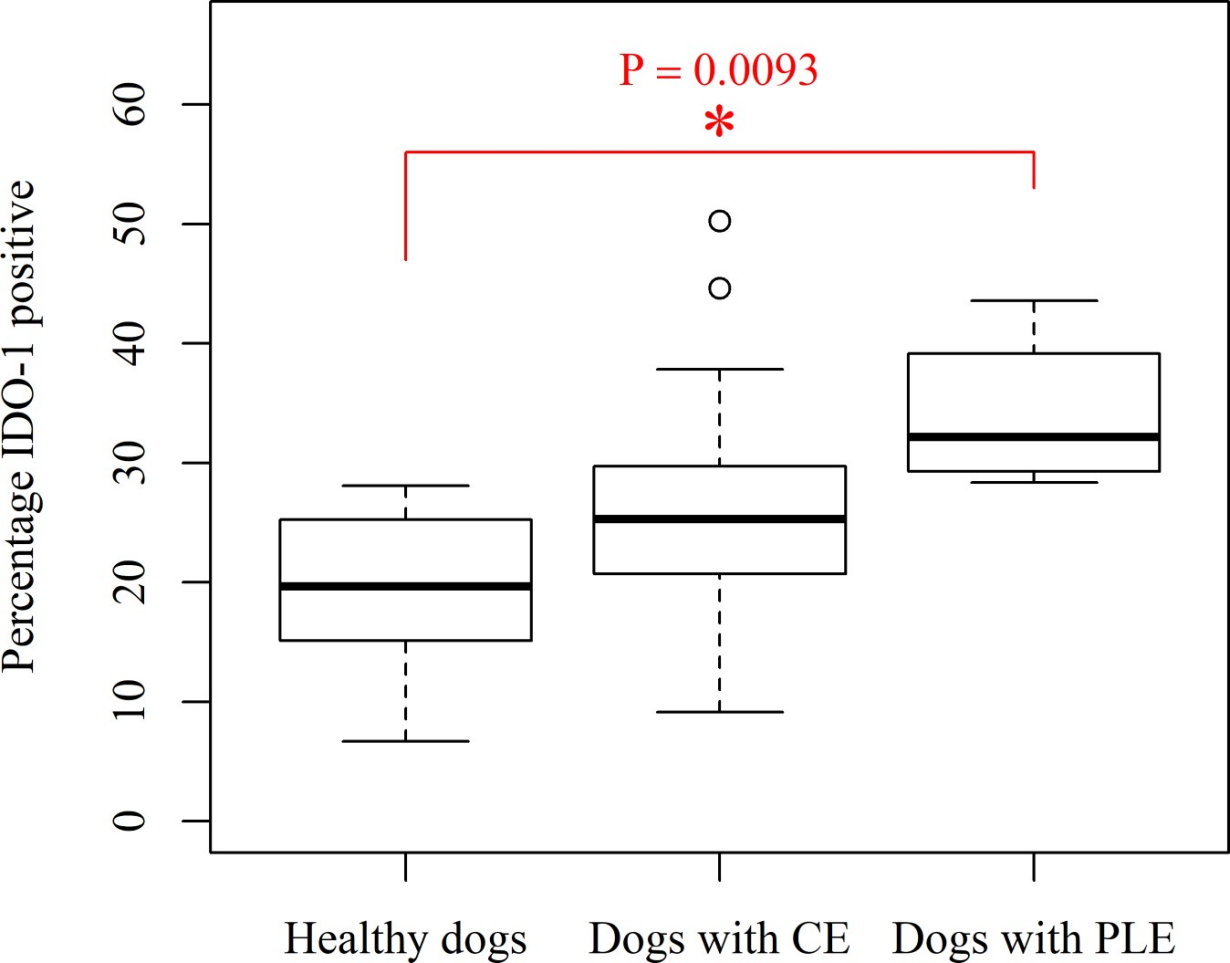
Box and whisker plot of percentage mucosal IDO-1 positive mRNA in the duodenum of dogs with protein-losing enteropathy (PLE), chronic enteropathy (CE) and healthy Beagle dogs. Dogs with PLE– 6 dogs, median– 32.18, interquartile range- 11.19. Dogs with CE– 18 dogs, median– 25.28, interquartile range- 9.56, healthy Beagle control dogs- 8 dogs, median– 19.65, interquartile range- 11.44.

**Fig 2:**
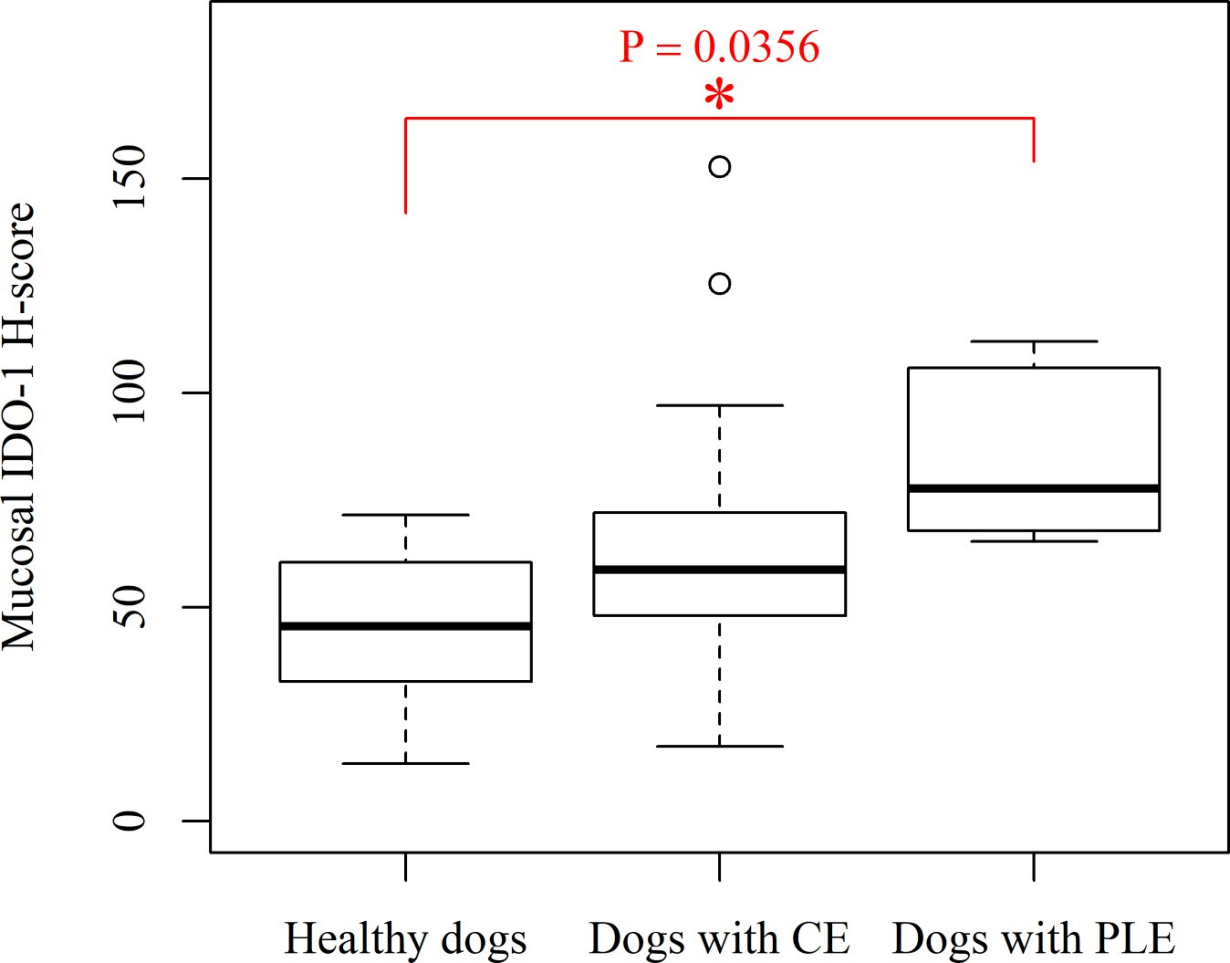
Box and whisker plot of mucosal IDO-1 H-score in the duodenum of dogs with protein-losing enteropathy (PLE), chronic enteropathy (CE) and healthy Beagle dogs. Dogs with PLE– 6 dogs, median– 77.66, interquartile range- 40.18. Dogs with CE– 18 dogs, median– 58.69, interquartile range- 26.82, healthy Beagle control dogs- 8 dogs, median– 45.26, interquartile range- 31.68.

**Fig 3:**
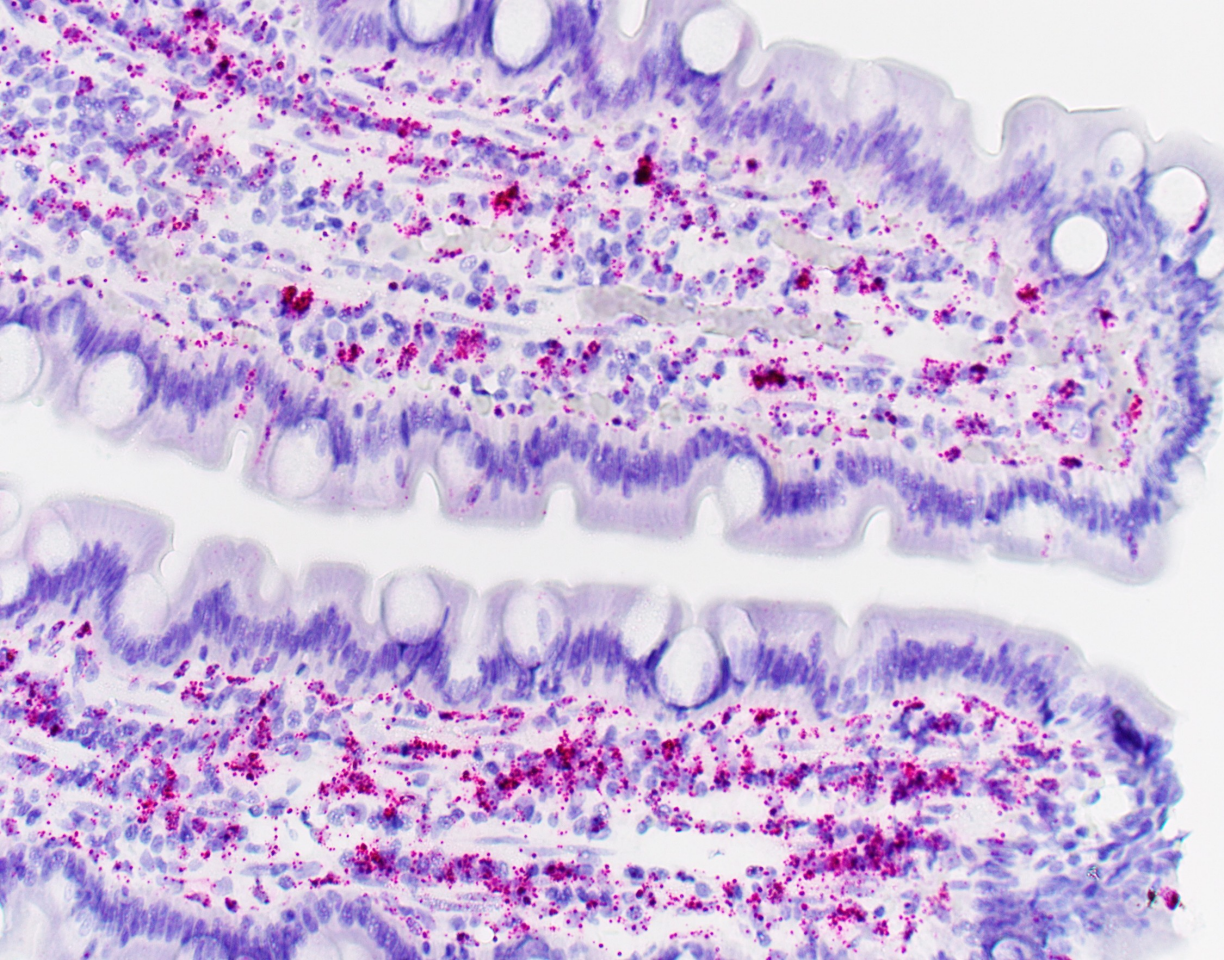
RNA *in situ* hybridization of IDO-1 mRNA in the duodenal mucosa of a dog with protein-losing enteropathy.

**Fig 4:**
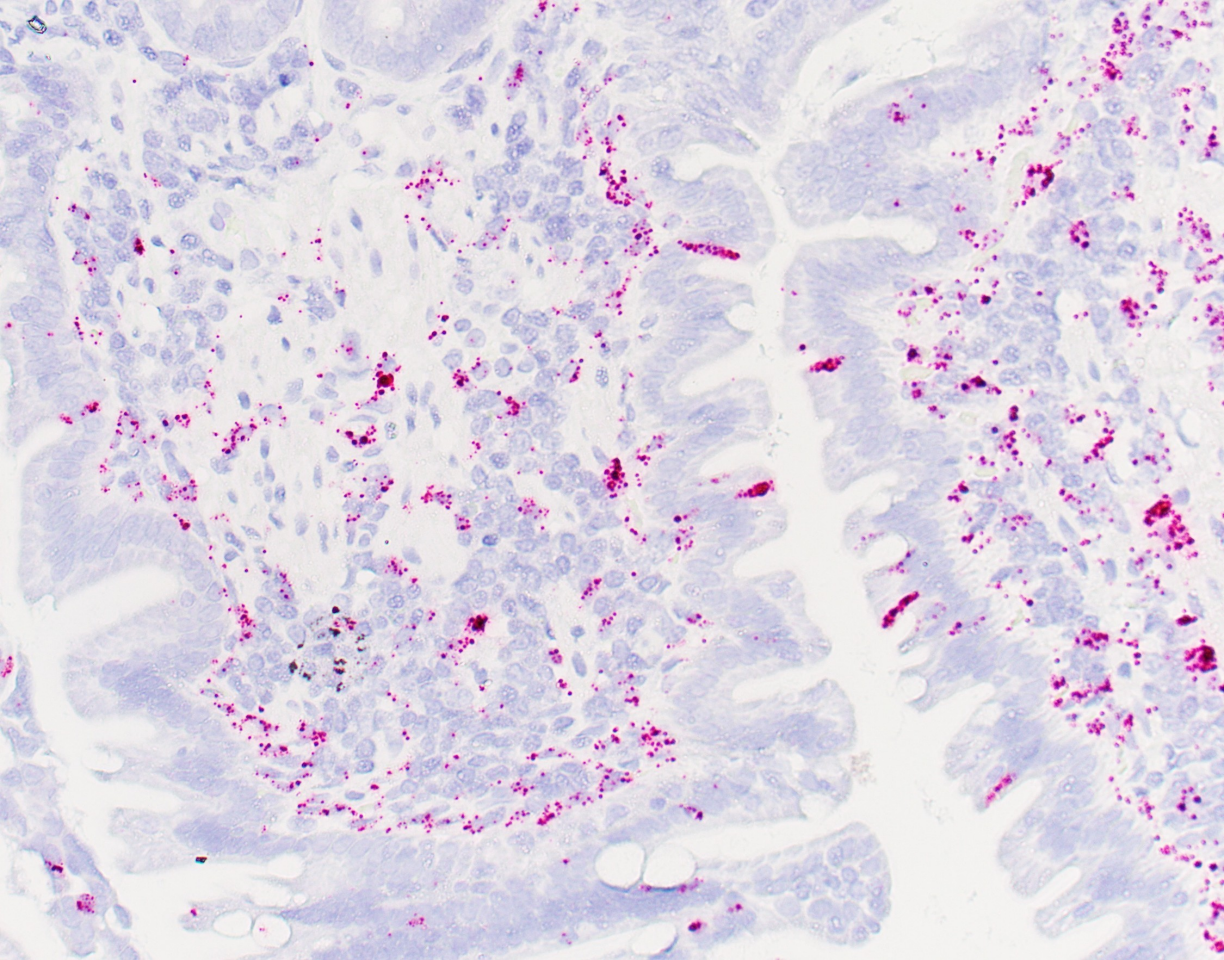
RNA *in situ* hybridization of IDO-1 mRNA in the duodenal mucosa of a healthy Beagle control dog.

Tukey's post-hoc analysis revealed that dogs with PLE have significantly higher mucosal IDO-1 percentage positive mRNA and mucosal IDO-1 H-score in the duodenum compared to healthy Beagle dogs (IDO-1 percentage positive mRNA: *P* = 0.0093, mean (± standard deviation); PLE– 34.12 (± 5.98); Control—19.36 (± 7.08); mucosal IDO-1 H-score; *P* = 0.0356, PLE– 84.37 (± 19.86); Control– 45.26 (± 19.33); Table 1 and Figs 1, 2, 3 and 4). There were no significant differences for the remainder of the 2 parameters between dogs with PLE and healthy Beagle dogs and for none of the 4 parameters between dogs with PLE and CE and between dogs with CE and healthy Beagle dogs (Table 1).

**Table 1:** *Post hoc* analysis of IDO-1 mRNA expression in the duodenal mucosa of dogs with protein-losing enteropathy (PLE), chronic enteropathy (CE), and healthy Beagle control dogs using RNA *in situ* hybridization. P-values obtained from Tukey's HSD (Honestly Significant Difference) post-hoc analysis after one-way analysis of variance testing of percentage mucosal IDO-1 positive mRNA and IDO-1 H-score in the duodenal mucosa of dogs with PLE (n = 6), dogs with CE (n = 18) and healthy Beagle control dogs (n = 8).

	Group comparisons	Difference in means	95% confidence interval for mean difference	Adjusted p-value (Tukey's HSD)
Percentage mucosal IDO-1 positive	CE—Healthy	7.364	(-1.637, 16.365)	0.1253
	PLE—Healthy	14.768	(3.327, 26.208)	0.0093*
	PLE—CE	7.404	(-2.582, 17.390)	0.1774
Mucosal IDO-1 H-score	CE—Healthy	20.104	(-8.859, 49.066)	0.2171
	PLE—Healthy	39.105	(2.294, 75.916)	0.0356*
	PLE—CE	19.001	(-13.130, 51.133)	0.3243

Significance was defined as *P* < 0.05^*^.

### Correlation between duodenal mucosal IDO-1 mRNA and serum tryptophan concentrations in dogs with PLE

**Fig 5:**
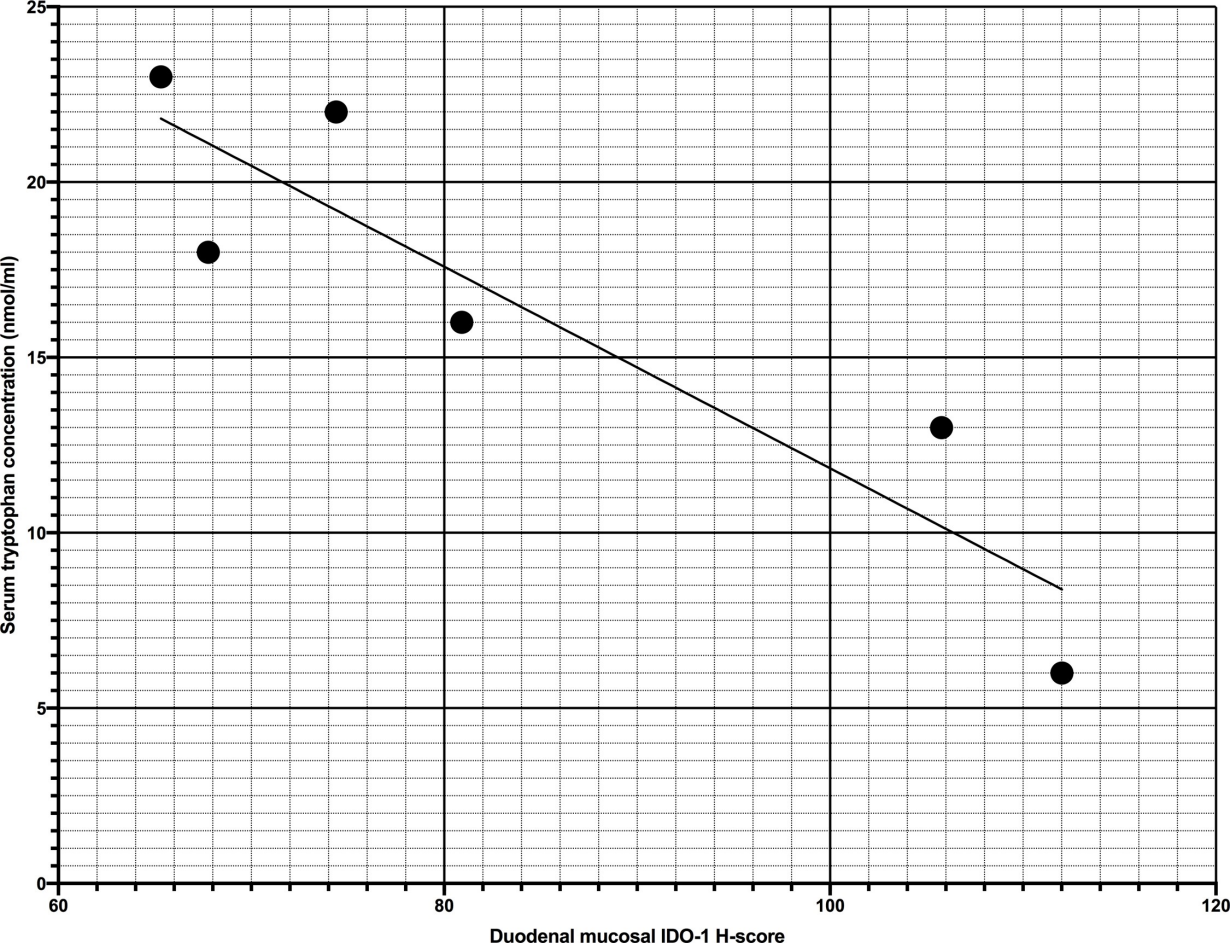
Scatter plot of serum tryptophan concentrations and duodenal mucosal IDO-1 H-score in dogs with protein-losing enteropathy. Scatter plot of serum tryptophan concentrations (nmol/ml) and duodenal mucosal IDO-1 H-score of 6 dogs with protein-losing enteropathy (PLE). Serum tryptophan concentrations were significantly negatively correlated with mucosal IDO-1 H-score in dogs with PLE (Spearman’s rank correlation co-efficient: -0.94, *P* = 0.0048).

The mRNA expression of IDO-1 in the duodenal mucosa was negatively correlated with serum tryptophan concentrations in dogs with PLE (average mucosal IDO-1 copies per cell: Spearman’s rank correlation coefficient (SRCC) = -0.94, *P* = 0.0048, average mucosal IDO-1 area per cell: SRCC = -0.89, *P* = 0.019, percentage mucosal IDO-1 positive: SRCC = -0.83, p = 0.042, mucosal IDO-1 H-score: SRCC = -0.94, *P* = 0.0048; Fig 5).

## Discussion

In this study, we show that dogs with PLE have significantly higher percentage mucosal IDO-1 positive mRNA and mucosal IDO-1 H-score in the duodenum compared to healthy Beagle control dogs. IDO-1 is the first and rate-limiting step in the catabolism of tryptophan in the kynurenine pathway [17, 18]. Through the catabolism of tryptophan and the generation of kynurenine metabolites, IDO-1 plays an important role in mucosal immune tolerance [19]. IDO-1 activity promotes depletion of tryptophan in the microenvironment, which induces cell cycle arrest of T-cells and increases their apoptosis [20]. However, recent evidence suggests that in a normal environment containing fatty acids, the immunosuppressive effect of IDO might not be due to decreased T cell survival and proliferation, as IDO supplied the required energy for cell survival and proliferation by increasing fatty acid oxidation [21]. Nevertheless, the catabolism of tryptophan via IDO-1 also leads to the generation of immune-modulating kynurenine metabolites [22]. These metabolites induce tolerogenic dendritic cells as well as T regulatory cells [23–25]. Kynurenine also selectively activates the transcription of aryl hydrocarbon, which results in the differentiation of CD4+ T cell into immunosuppressive T regulatory cells [26]. IDO- 1 also possesses a non-enzymatic function that contributes to TGF-beta led tolerance [27]. Therefore, IDO-1 expressing antigen presenting cells are crucial in suppressing pro-inflammatory mucosal T-cells that drive chronic intestinal inflammation. Epithelial IDO-1 activity may also function to limit microbial invasion as IL-27 has been shown to inhibit growth of intestinal bacteria and promote epithelial barrier protection via induction of IDO-1 in human and mouse intestinal epithelial cells [28]. Therefore, intestinal IDO-1 acts as a natural brake to inflammatory responses via the metabolism of tryptophan.

IDO-1 expression is stimulated by proinflammatory cytokines including interferon gamma, tumor necrosis factor-alpha and interleukin-1 beta and activation of toll-like receptors [2, 29, 30]. In line with this notion, IDO-1 is one of the most up-regulated genes in human IBD and animal models of colitis [4, 31, 32]. Also, several studies have documented increased intestinal IDO-1 mRNA and protein in human and animal models of IBD [6, 33, 34]. Consistent with these earlier studies, our results also showed increased IDO-1 mRNA in the duodenal mucosa of dogs with PLE compared to healthy Beagle dogs. This upregulation of duodenal IDO-1 mRNA in dogs with PLE is likely reflective of increased inflammation and immune activation within the duodenum. Interestingly, our study did not document a difference in IDO-1 mRNA expression in the duodenal mucosa between dogs with CE and healthy Beagle control dogs. There could be a number of possibilities for this finding. First, our study used a relatively small number of dogs, which could have reduced the statistical power of the analysis; second, the immunological pathogenesis of CE and PLE may be different, which could explain the lack of a significant difference in dogs with CE; and third, most of the dogs with CE in our study were German shepherd dogs which may have breed-specific patterns of cytokine expression, as suggested by recent investigations on the role of Th2 cytokines in the pathogenesis of CE in this breed [35]. Interestingly, one study showed that exposure of peripheral blood mononuclear cells to Th2 cytokines resulted in higher tryptophan concentrations as the breakdown of this amino acid was suppressed [36]. Th2 cytokines counteract interferon-gamma and other Th1 mediated cytokines and therefore this may result in lower IDO-1 expression. Therefore, as nearly half of the dogs in the CE group were German shepherd dogs, which may have a Th2 driven inflammatory process for their CE, this might potentially explain why dogs with CE in our study did not have significantly increased IDO-1 mRNA expression compared to healthy controls. However, further studies are needed to determine which specific proinflammatory cytokine(s) promote increased IDO-1 mRNA expression in dogs with PLE.

Although, IDO-1 is increased in the intestinal tract of humans and mouse models of IBD, studies suggest a beneficial role for this upregulation. IDO-1 knockout mice exhibit worse colitis, T-cell infiltration and mortality [37]. Also, disease severity was worsened in mice with trinitrobenzenesulfonic acid induced colitis receiving IDO-1 inhibition [5]. On the other hand, induction of IDO-1 reduced colon injury and ameliorated lethality in graft-*versus*-host disease in mice [38]. Collectively, these findings suggest that IDO-1 functions as a negative feedback mechanism to limit the development of chronic inflammation. Therefore, the increased IDO-1 expression likely represents an anti-inflammatory mechanism to counterbalance the pro-inflammatory milieu causing intestinal inflammation. Hence, the expression of IDO-1 could be an indicator of the level of intestinal inflammation due to this counter-regulatory response. Unfortunately, due to the small number of dogs in our study, correlations with severity of duodenal histopathology or clinical disease activity could not be performed to determine if duodenal IDO-1 mRNA expression increases with disease severity in dogs with PLE. Future studies should focus on determining if such correlations exist, as intestinal IDO-1 mRNA expression could then be used as a marker of clinical and histological severity in dogs with PLE.

Increased IDO-1 expression in the intestinal mucosa also decreases serum tryptophan concentrations and increases kynurenine concentrations in patients with Crohn’s disease a type of IBD in humans [8]. Our study showed similar findings of a negative correlation between duodenal IDO-1 mRNA expression and serum tryptophan concentration in dogs with PLE. We have previously shown that dogs with PLE have significantly lower serum tryptophan concentrations compared to healthy dogs [9]. Results from our current study suggest that decreased serum tryptophan concentrations in dogs with PLE might be due to accelerated catabolism of this amino acid from increased intestinal IDO-1 expression. The kynurenine-to-tryptophan ratio, which is an indicator of IDO-1 activation in the intestinal tract, has been shown to be negatively correlated with Crohn’s disease activity [8]. Unfortunately, due to the small number of PLE dogs included in our study and the absence of serum kynurenine concentration measurements in these dogs, similar conclusions could not be made with regards to clinical disease activity. However, further studies should assess whether serum concentrations of tryptophan and kynurenine metabolites could serve as a useful biomarker of intestinal mucosal inflammation and immune activation in dogs with PLE.

Another explanation for a negative correlation between serum tryptophan concentration and duodenal IDO-1 mRNA expression in dogs with PLE could be due to increased IDO-1 mRNA expression from worsening intestinal inflammation leading to increased enteric albumin loss, which could lead to reduced serum tryptophan concentrations. However, our study measured only free tryptophan in serum and therefore is unlikely to have been directly affected by the serum albumin concentrations. Also, serum concentrations of free tryptophan in humans with chronic renal failure were shown to be uninfluenced by serum protein concentrations and the free fraction was normal in those patients with hypoalbuminemia [39]. Therefore, increased enteric albumin loss is unlikely to be a cause for the negative correlation between duodenal IDO-1 mRNA expression and serum tryptophan concentration in dogs with PLE. Similarly, we have previously documented that appetite (anorexic, hyporexic, unchanged or increased) did not significantly affect serum tryptophan concentrations in dogs with PLE [9].

Dogs with PLE can have poor prognosis following failure with immunomodulatory therapy [40]. Pharmacologic agents, which inhibit or potentiate IDO-1 expression and activity, may have the potential as treatment for dogs with PLE. However, upregulation of IDO-1 might function as a regulatory mechanism to suppress immune response and protect intestinal mucosal tissue [6, 34]. Therefore, further studies are needed to determine the role of increased IDO-1 expression in dogs with PLE before any recommendations can be made regarding inhibiting or potentiating this enzyme in the potential treatment of this disease.

Potential limitations of our study include the use of partial thickness endoscopic biopsies for RNA ISH rather than full-thickness biopsies, possibly missing cells expressing IDO-1 mRNA in the deeper mucosa. In addition, our study exclusively assessed mRNA levels and provides no information on protein expression *per se*. In other words, the production of the IDO-1 protein could have been unaltered despite the increase in mRNA expression. Additionally, our study only included mRNA expression data from the duodenum, such that expression in other intestinal segments where tryptophan absorption may occur e.g. the ileum may have shown different results. Finally, dogs with CE in our study did not have serum tryptophan concentrations assessed, therefore unlike the PLE group, correlations regarding serum tryptophan concentration and duodenal mucosal IDO-1 mRNA expression could not be performed.

In conclusion, our study demonstrates that dogs with PLE have increased mRNA expression of IDO-1 in the duodenal mucosa compared to healthy Beagle dogs and this negatively correlated with serum tryptophan concentrations. Further studies are needed to determine the role of increased IDO-1 expression in the duodenum of dogs with PLE, as well as the inflammatory pathways responsible for the increased expression of IDO-1.

## Supporting information

S1 File Excel sheet of IDO-1 mRNA expression in the duodenal mucosa of all dogs. Results of the average mucosal IDO-1 copies per cell, average mucosal IDO-1 area per cell, percentage mucosal IDO-1 positive and mucosal IDO-1 H-score for all dogs included in the study and serum tryptophan concentrations for the 6 dogs diagnosed with PLE.
(XLSX)
